# Identifying Candidate Genes for Short Gestation Length Trait in Chinese Qingping Pigs by Whole-Genome Resequencing and RNA Sequencing

**DOI:** 10.3389/fgene.2022.857705

**Published:** 2022-05-16

**Authors:** Zezhang Liu, Jun Yang, Hong Li, Zhuxia Zhong, Jian Huang, Jie Fu, Hucheng Zhao, Xiaolei Liu, Siwen Jiang

**Affiliations:** ^1^ Key Laboratory of Swine Genetics and Breeding of the Agricultural Ministry and Key Laboratory of Agricultural Animal Genetics, Breeding and Reproduction of the Ministry of Education, College of Animal Science and Technology, Huazhong Agricultural University, Wuhan, China; ^2^ College of Animal Science, Yangtze University, Jingzhou, China; ^3^ Novogene Bioinformatics Institute, Beijing, China; ^4^ Shenzhen Institute of Nutrition and Health, Huazhong Agricultural University Hubei Hongshan Laboratory, Wuhan, China; ^5^ Shenzhen Branch, Guangdong Laboratory for Lingnan Modern Agriculture, Genome Analysis Laboratory of the Ministry of Agriculture, Agricultural Genomics Institute at Shenzhen, Chinese Academy of Agricultural Sciences, Shenzhen, China; ^6^ The Cooperative Innovation Center for Sustainable Pig Production, Wuhan, China

**Keywords:** sequencing, short gestation length, selective sweep, RNA-seq, EGFR

## Abstract

Gestation length is a complex polygenic trait that affects pig fetal development. The Qingping (QP) pig, a Chinese native black pig breed, is characterized by short gestation length. However, the genetic architecture of short gestation length is still not clear. The present study aimed to explore the genetic architecture of short gestation length in QP pigs. In this study, selective sweep analyses were performed to detect selective sweep signatures for short gestation length traits between 100 QP pigs and 219 pigs from 15 other breeds. In addition, differentially expressed genes for the short gestation length between QP pigs and Large White pigs were detected by RNA sequencing. Comparing candidate genes from these methods with known genes for preterm birth in the database, we obtained 111 candidate genes that were known preterm birth genes. Prioritizing other candidate genes, 839 novel prioritized candidate genes were found to have significant functional similarity to preterm birth genes. In particular, we highlighted *EGFR*, which was the most prioritized novel candidate relative to preterm birth genes. Experimental validations in placental and porcine trophectoderm cells suggest that *EGFR* is highly expressed in the QP pigs with short gestation length and could regulate the NF-κΒ pathway and downstream expression of *PTGS2*. These findings comprehensively identified candidate genes for short gestation length trait at the genomic and transcriptomic levels. These candidate genes provide an important new resource for further investigation and genetic improvement of gestation length.

## Introduction

Reproductive traits play key roles in the reproductive efficiency of pigs and economic profit. In addition to some well-known reproductive traits, including total number born, number born alive, litter birth weight, and average birth weight, gestation length is also a key aspect of reproduction. The gestation length of pigs is well documented as 3 months, 3 weeks, and 3 days (114 d). A shorter gestation length with spontaneous parturition results in a higher reproductive efficiency of pigs. Here, we introduced a Chinese native black pig breed with a short gestation length. The Qingping (QP) pig was characterized as a pig breed with short gestation length in *Animal Genetic Resources in China-Pigs*, which reported that the average gestation length was 111.51 d. Based on this information, the QP pig has the potential to play an important role in the genetic improvement of gestation length. However, traditional breeding technologies are limited with regard to significant genetic improvement in gestation length because gestation length is not measurable until sexual maturity. Thus, it is important to clearly understand the genetic architecture of short gestation length. Accordingly, quantitative trait loci (QTLs) for gestation length of pigs have been identified in association studies using microsatellite markers and the PorcineSNP60 BeadChip (Wilkie et al., 1999; Chen et al., 2010; Onteru et al., 2012; Hidalgo et al., 2016). Although our previous study reported differentially expressed genes in the fetal membranes and placenta between pigs with short gestation length and normal gestation length during parturition (Li et al., 2015), the genetic architecture of short gestation length is still largely unclear.

Pregnancy and parturition are key factors in reproduction, and gestation length is calculated based on the timing of conception and parturition. The process of parturition is a choreographed sequence of events and converges on the important pathways in term and preterm birth (Golightly et al., 2011; Romero et al., 2014). One convincing hypothesis is that fetal membrane senescence is the initiator of parturition and that a timely and safe birth is facilitated by the convergence and integration of processes involved in linking senescence, endocrine, inflammatory, and physical factors (Menon et al., 2016). Inflammation is essential for successful delivery, and untimely inflammatory triggers are involved in adverse pregnancy outcomes, including preterm birth (Nadeau-Vallee et al., 2016). The proinflammatory phenotype is induced by activating the NF-κB pathway and downstream expression of *PTGS2* in the myometrium and cervix (Renthal et al., 2013). Our previous study indicated that miR-144 inhibited preterm birth by repressing the expression of *PTGS2* (Li et al., 2016). In humans, short gestation length has been termed spontaneous preterm birth (<37 weeks of gestation). Preterm birth is the leading cause of health risks for newborns and severe childhood neurological disabilities (Liu et al., 2012). Unlike human preterm birth, a short gestation trait might be caused by genetic differences between Qingping pigs and other breeds and do not cause piglet development and health problems. The common characteristic between a short gestation length and spontaneous preterm birth is the early initiation of parturition without artificial induction. Substantial evidence indicates that genetic factors contribute to the risk of preterm birth during pregnancy (Bezold et al., 2013). Therefore, investigations of preterm birth can provide useful insights with regard to short gestation length. To understand how genetic factors contribute to the risk of preterm birth, genomic and transcriptomic analyses have been applied in many preterm birth studies (Lackritz et al., 2013). The robust genetic associations for term or preterm birth were determined by a genome-wide association study (GWAS) in 43,568 women (Zhang et al., 2017). RNA sequencing is also an indispensable tool that identifies transcriptome-wide differentially expressed genes between term and preterm birth (Enquobahrie et al., 2009; Weiner et al., 2010; Heng et al., 2014).

Long-term natural and artificial selection ultimately result in phenotypic differences. Selective sweep regions can also explain phenotypic differences among breeds within a species. Several studies have used different strategies to explore selective sweep regions for different traits among breeds (Cao et al., 2016; Lu et al., 2016). Notably, Ma et al. indicated that selective sweep regions are always very broad-spectrum, including the genetic architecture of many traits. Therefore, they defined trait-specific selective sweep regions using a selective sweep analysis of phenotypic gradients across different groups. Using this strategy, they successfully identified trait-specific candidate genes for backfat thickness (Ma et al., 2019). These results suggested that phenotypic gradients across different groups might also be applied to detect selective regions for short gestation length.

The purpose of the current study was to determine the genetic architecture of short gestation length. An integrative method was applied to determine candidate genes for short gestation length at the genomic and transcriptomic levels. We sequenced the genomes of 100 QP pigs and downloaded 219 published pig sequence datasets, thus enabling us to identify high-density SNPs. We conducted selective sweep analyses based on high-density SNPs for short gestation length in these 319 pigs. A differential expression analysis was performed with RNA-seq data from 12 QP pigs and six Large White (LW) pigs. *EGFR* was verified in the placenta and porcine trophectoderm (pTr) cells.

## Materials and Methods

### Sample Preparation and Whole-Genome Resequencing

All samples were collected from the Qingping pig conservation farm in Yichang, Hubei, China. Ear tissues were collected, frozen in liquid nitrogen, and stored at −80°C until needed. Genomic DNA was extracted using a standard phenol/chloroform method. Sequencing was performed to generate 150-bp paired-end reads using the HiSeq 4,000 platform (Illumina). Raw reads underwent quality control procedures to remove the lower quality reads. Additionally, we downloaded the published whole-genome sequencing data of 219 pigs from 15 breeds.

### Phenotype

The gestation lengths were recorded for all 100 QP pigs in this study. In total, we collected 398 gestation lengths from these 100 QP pigs, which were approximately normally distributed (Supplementary Table S1). The average gestation lengths of QP pigs in this study ranged from 110 to 117 d; 73% of values were less than 114 d. The phenotypes of the other 15 breeds were reported as the average gestation length (Supplementary Table S2).

### Sequence Alignment and Variant Calling

Clean reads from all individuals were aligned to the *Sscrofa11.1* reference genome using the Burrows–Wheeler Aligner (BWA) (Li and Durbin, 2009). SNPs were genotyped and filtered using Genome Analysis Toolkit (GATK) packages. The SNPs were further filtered using VCFtools v0.1.16 and PLINK v2.0 (Danecek et al., 2011; Chang et al., 2015). The filtered SNPs were imputed using Beagle (Browning et al., 2018). Finally, high-quality common SNPs of 319 pigs were produced by PLINK v2.0.

### Linkage Disequilibrium Analysis

Linkage disequilibrium (LD) decay analysis was performed using PopLDdecay based on variant call format files (Zhang et al., 2019). The average *r* ^2^ value was calculated for every pair of SNPs with a maximum distance of 300 kb between two SNPs and was averaged across the whole genome. The LD decay figure was drawn using R.

### Population Structure Analyses

High-quality common SNPs with an *r* ^2^ < 0.2 between every pair of SNPs were extracted by PLINK v2.0. A total of 689,158 SNPs in VCF format remained and were converted to a PHYLIP file using vcf2phylip for phylogenetic analysis. We subsequently constructed a neighbor-joining (NJ) tree using PHYLIP and visualized the NJ tree using the ggtree package in R (Yu et al., 2016). Principal component analysis (PCA) was performed using PLINK v2.0 with high-quality common SNPs. PC1 and PC2 were visualized using the ggplot2 package in R.

### Detection of Selective Sweeps

To detect the short gestation length selection signatures, we produced three phenotypic gradient QP groups to compare with the other 219 pigs. The basic steps were as follows: 1) ranking the phenotypic value (average gestation length) of 100 QP pigs (QP) and 219 other pigs, which were recorded as the first population pair; 2) based on the first step, 73 QP pigs with shorter gestation length ( < 114  d, QP114) were selected from the 100 QP pigs, and the QP114 group and 219 other pigs were recorded as the second population pair; 3) based on the second step, 30 QP pigs with a shorter gestation length ( < 113  d, QP113) were selected from the 73 QP pigs, and the QP113 group and 219 other pigs were recorded as the third population pair. We performed selective sweep analyses on these three population pairs using three approaches based on high-quality common SNPs: *π* ratios and *F*_ST_ calculated using VCFtools v0.1.16 and the cross-population extended haplotype homozygosity (XP-EHH) approach using the rehh package in R (Danecek et al., 2011; Gautier et al., 2017). Values of *π* were calculated using a 10-kb sliding window and a 10-kb sliding step. We calculated *π*QP113/*π*others, *π*QP114/*π*others, and *π*QP/*π*others ratios and selected the top 1% absolute *π* ratios as candidate selective sweeps. The *F*_ST_ values were also calculated with a 10-kb sliding window and a 10-kb sliding step, and the top 1% windows were chosen as selective sweeps. For the XP-EHH approach, unstandardized XP-EHH values were calculated, and the two-way top 1% unstandardized XP-EHH value SNPs were selected as candidate loci. The *F*_ST_, *π* ratios, and XP-EHH selection signatures that exhibited a gradient change were defined as specific signatures for short gestation length. Functional genes in the *F*_ST_ and *π* ratio candidate windows were annotated using the biomaRt package in R based on *Sscrofa11.1*. The functional genes of XP-EHH were annotated using ANNOVAR based on *Sscrofa11.1.*

### RNA-Seq

We analyzed the transcriptome of six QP pigs with short gestation length (112 d), six QP pigs with normal gestation length (114 d), and six LW pigs with normal gestation length (114 d) as follows: 1) short gestation length QP pigs at late pregnancy (109 d, PRE109Q; n = 3), 2) short gestation length QP pigs at parturition (111 d, PAR111Q; n = 3), (3) normal gestation length QP pigs at late pregnancy (111 d, PRE111Q; n = 3), 4) normal gestation length QP pigs at parturition (114 d, PAR114Q; n = 3), 5) normal gestation length LW pigs at late pregnancy (112 d, PRE112D; n = 3), and 6) normal gestation length LW pigs at parturition (114 d, PAR114D; n = 3). The placental tissues from pigs in the PRE109Q and PRE111Q groups were dissected and collected immediately after slaughter. Total RNA was isolated using TRIzol, and Illumina libraries were constructed. RNA sequencing (RNA-seq) was performed on an Illumina HiSeq 4,000 platform (Illumina) using a paired-end approach with 150-bp reads. The RNA-seq data analysis of samples from pigs in the PAR114Q, PAR111Q, PRE112D, and PAR114D groups was performed on an Illumina HiSeq 2000 platform (Illumina) using a paired-end approach with 100-bp reads in our former study (Li et al., 2015).

Raw sequence read files were first quality checked by FastQC software. Clean reads were mapped to the *Sscrofa11.1* reference genome using TopHat2 (Kim et al., 2013). DESeq2 was used to quantify gene expression differences in terms of fold change (log2) and statistical significance (Benjamini–Hochberg-corrected *p* value) (Love et al., 2014). Genes were annotated as differentially expressed at a fold change >2 and an adjusted *p* < 0.001. We excluded differentially expressed genes with different gestation statuses. First, we detected differentially expressed genes between late pregnancy and parturition in short gestation length QP pigs and LW pigs. Combining differentially expressed genes, we defined these genes as differentially expressed due to different gestation statuses. Second, we performed pairwise differentially expressed comparisons, PRE112D vs. PRE109Q and PAR114D vs. PAR111Q, to identify differentially expressed genes for short gestation length. Finally, we excluded differentially expressed genes with different gestation statuses.

### Functional Enrichment Analyses

Candidate gene enrichment analyses were performed using the clusterProfiler package in R (Yu et al., 2012). Gene Ontology (GO) and KEGG pathway enrichment were performed based on genome-wide annotation for Pig (org.Ss.eg.db).

### Cell Culture

Mononuclear pTr cells were obtained from Jian Peng’s lab in the College of Animal Science and Technology, Huazhong Agricultural University. We cultured pTr cells in a 25-cm^2^ cell culture flask using DMEM/F-12 (1:1) basic culture medium (Gibco) supplemented with 10% fetal bovine serum (FBS). For verification experiments, pTr cells were grown in a culture medium to 80% confluence in 6-well cell culture plates. The cells were cultured for 24 h with serum-free DMEM/F-12 and then treated with recombinant human EGF (100 ng/ml, 236-EG R&D) for 0, 5, 15, 30, 60, or 120 min. The dose of EGF was described in a previous study (Jeong et al., 2013).

### Small Interfering RNA (siRNA) Transfection

For the messenger RNA sequences of pig *EGFR*, a negative control (5′-UUC UCC GAA CGU GUC ACG UTT-3′) and three potential small interfering RNA target sites (si_EGFR_135, forward 5′-GCC UCC AGA GGA UGU UCA ATT-3′ and reverse 5′-UUG AAC AUC CUC UGG AGG CTT-3’; si_EGFR_298, forward 5′-GCA GAU CAU CCG AGG AAA UTT-3′ and reverse 5′-AUU UCC UCG GAU CUG CTT-3’; and si_EGFR_495, forward 5′-GCG ACU UUC UAA GCA ACA UTT-3′ and reverse 5′-AUG UUG CUU AGA AAG UCG CTT-3′) were synthesized by GenePharma. Cells were cultured in the culture medium to 60–70% confluence at the time of transfection. Transfections were performed using a negative control or siRNAs at a final concentration of 100 nM using Lipofectamine RNAiMAX (Invitrogen) in Opti-MEM (Gibco) for 6 h. Downregulation of the EGFR expression was tested using real-time PCR and Western blot after 48 h of transfection. The effective siRNA (si_EGFR_135) was used for further experiments. Furthermore, cells were cultured for 24 h with serum-free DMEM/F-12 after 24 h of transfection using si_EGFR_135, and then, the EGF treatments were performed as described previously.

### RNA Extraction and Real-Time PCR

We extracted RNA from 18 placental tissues (12 QP pigs and six LW pigs) and pTr cells after 48 h of transfection (negative control or siRNAs) using RNAiso Plus (9,109, TaKaRa). The RNA concentration was measured using a NanoDrop 2000 spectrophotometer (Thermo Scientific) based on the absorbance at 260 nm. The isolated RNA was reverse transcribed into cDNA for real-time PCR using the PrimeScript RT reagent Kit with gDNA Eraser (RR047A, TaKaRa). Real-time PCR was performed using a QuantiNova SYBR Green PCR Kit (208054, QIAGEN) on a CFX384 Touch Real-Time PCR System (Bio-Rad). The real-time PCR conditions were 95 C for 5 min followed by 40 cycles at 95 C for 30 s, 58 C for 30 s, and 72 C for 15 s using a melting curve program (increasing the temperature from 56 to 95 C by 0.5 C per 10 s) and continuous fluorescence measurements. Relative quantification of the mRNA levels of *EGFR* in placental tissues was performed using the comparative Ct method.

### Western Blot Analyses

For placental tissues, we added 300 μl ice-cold RIPA lysis buffer (Beyotime) supplemented with protease inhibitor cocktail (1:100 dilution, B14001, Bimake) and phosphatase inhibitor cocktail (1:100 dilution, B15001, Bimake) to 20 mg of tissues and broken tissues using a Tissuelyser-24 L (JingXin, Shanghai, China). The extract was centrifuged at 14,000 rpm for 15 min at 4 C to remove tissue debris. The cells were washed twice with ice-cold PBS and harvested in ice-cold RIPA lysis buffer (Beyotime) supplemented with protease inhibitor cocktail (1:100 dilution, B14001, Bimake) and phosphatase inhibitor cocktail (1:100 dilution, B15001, Bimake). The extract was centrifuged at 14,000 rpm for 15 min at 4 C to remove cellular debris. Protein concentrations were determined using a Pierce BCA Protein Assay Kit (23225, Thermo Scientific). SDS-PAGE was used to further separate 20 μg of the protein sample, which was then transferred onto PVDF membranes (1620177, Bio-Rad). The membranes were blocked for 2 h in Tris-buffered saline containing 0.01% Tween 20 with 5% nonfat dried milk and then incubated overnight at 4 C with the appropriate primary antibodies after washing. After washing, the membranes were incubated with the secondary antibody for 2 h. Immunoreactive proteins were detected using a Clarity Western ECL Substrate Kit (1705061, Bio-Rad) after washing and were imaged using an ImageQuant LAS4000 mini system. The primary antibodies used for Western blotting were as follows: EGFR (1:5000 dilution, 18986-1-AP, Proteintech), PTGS2 (1:1000 dilution, 12282, CST), p-p65 (1:1000 dilution, Ser536, 3033, CST), and BACT (1:20000 dilution, AC004, ABclonal). The secondary antibodies used for Western blotting are as follows: HRP goat anti-rabbit IgG (1:5000 dilution, AS014, ABclonal) and HRP-labeled goat anti-mouse IgG (1:4,000 dilution, GB23301, Servicebio).

## Results

### SNP Calling and Phenotypes

**FIGURE 1:**
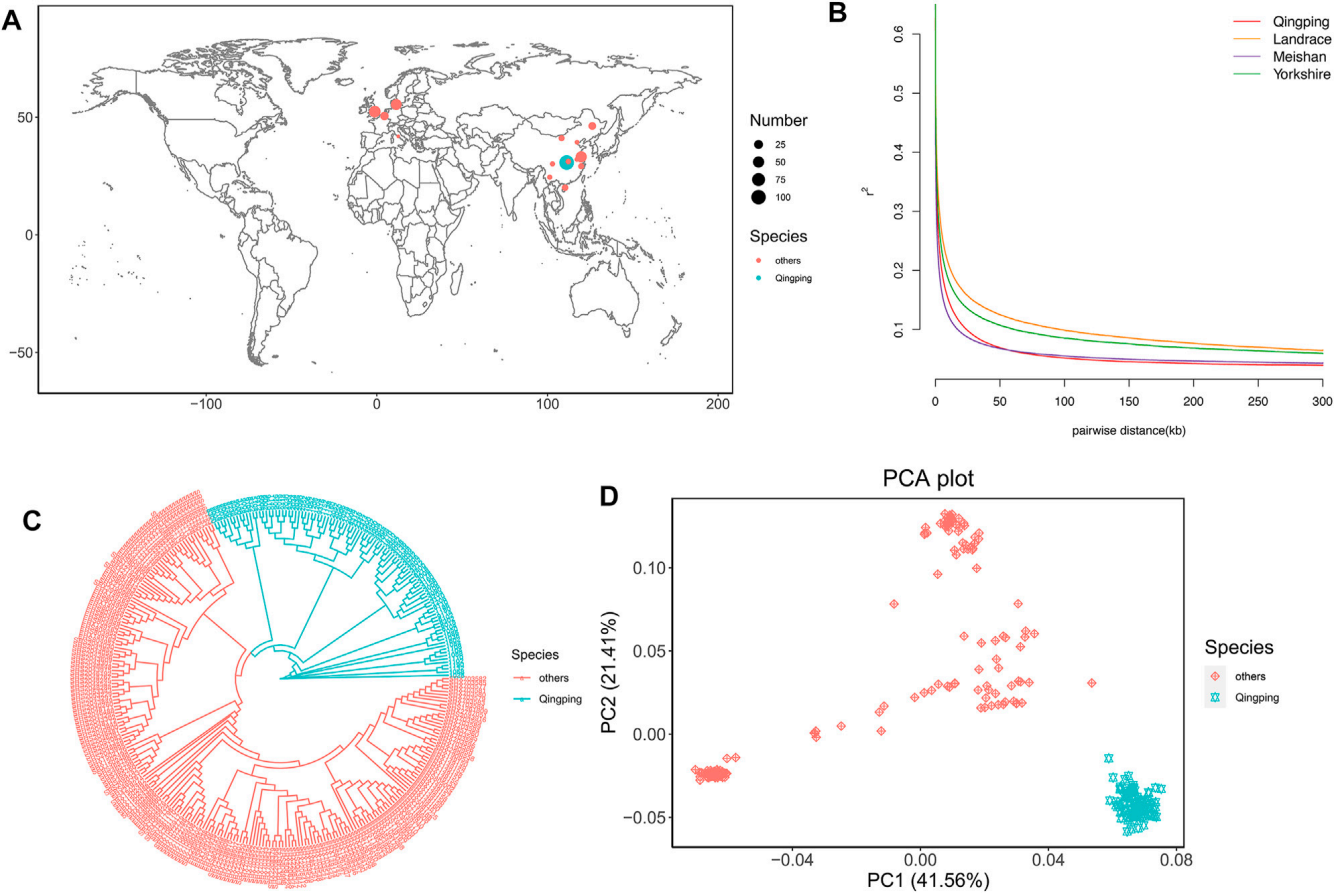
Population analyses. **(A)** Geographic distribution of the 319 pigs. QP pigs and 15 other breeds are represented by blue and pink dots on the world map, respectively. The sample size is represented by the dot size. **(B)** LD decay of QP and downloaded breeds. **(C)** NJ tree of the 319 pigs. QP pigs and 15 other breeds are represented by blue and pink lines, respectively. **(D)** Plots of PC1 and PC2 for the 319 pigs.

After performing whole-genome sequencing for 100 QP pigs, 2.76 TB of sequences with an average 98.47% coverage and an average 9.66-fold depth were generated (Supplementary Table S3). In addition, we downloaded previously published resequencing data from 219 pigs of 15 breeds (Supplementary Table S4 and Figure 1A). Genotyped SNPs were filtered to have a minor allele frequency (MAF) > 0.05, individual missing rate <10%, SNP call rate >90%, and Hardy–Weinberg equilibrium (HWE) test *P* > 1E−6. After filtering, 10,342,544 high-quality common SNPs were retained.

### Population Analysis

The LD decay showed that QP and Meishan had a smaller LD than the other breeds (Figure 1B). The phylogenetic tree showed that QP was clearly clustered alone and separate from the other breeds (Figure 1C). Additionally, PCA further confirmed the tree results (Figure 1D). PC1 and PC1 explained 41.56 and 21.41% of the variation, respectively. The QP pigs clustered together and showed high genetic distances from other breeds. These results support that the QP pig is an independent breed and suggest that short gestation length might be associated with genetic differences among QP and other breeds.

### Genomic Signatures Related to Selection

**FIGURE 2:**
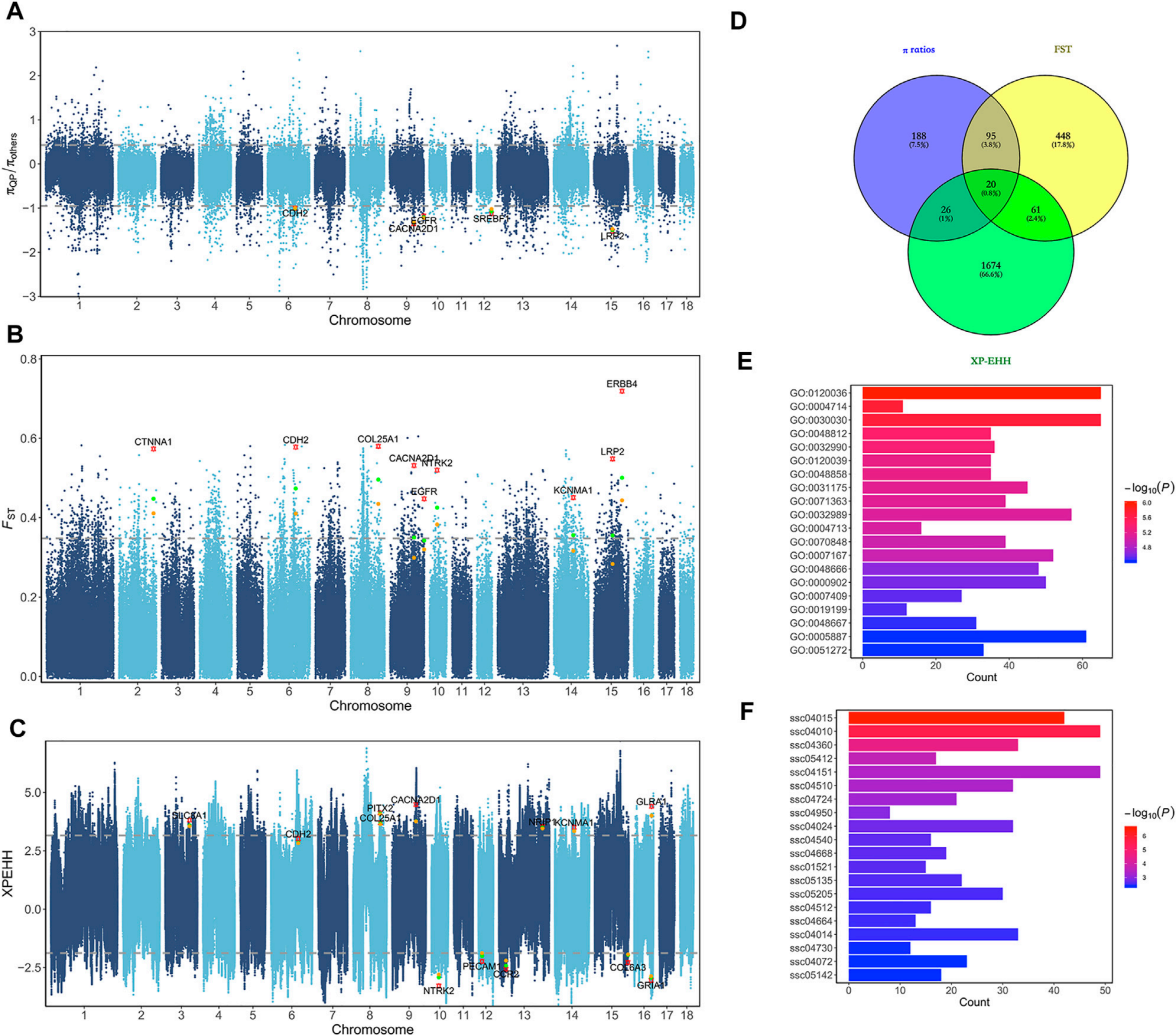
Selective sweep analyses for short gestation length. **(A)** Manhattan plot of *π* ratios between QP and other breeds. The *π* ratios were transformed using log10. **(B)** Manhattan plot of *F*_ST_ between QP and other breeds. **(C)** Manhattan plot of XP-EHH between QP and other breeds. **(D)** Venn diagram of selective candidate genes. **(E)** Bar plot of GO enrichment for selective candidate genes. **(F)** Bar plot of KEGG pathway enrichment for selective candidate genes.

**FIGURE 3:**
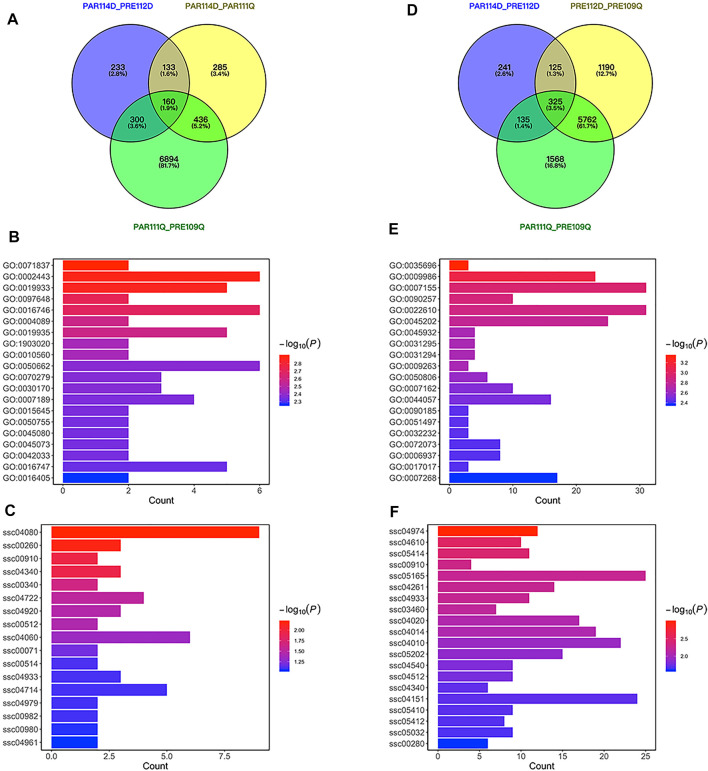
Differentially expressed genes for short gestation length. **(A)** VENN plot of differentially expressed genes for short gestation length in parturition. **(B)** Bar plot of GO enrichment for differentially expressed genes in parturition. **(C)** Bar plot of KEGG pathway enrichment for differentially expressed genes in parturition. **(D)** VENN plot of differentially expressed genes for short gestation length in late pregnancy. **(E)** Bar plot of GO enrichment for differentially expressed genes in late pregnancy. **(F)** Bar plot of KEGG pathway enrichment for differentially expressed genes in late pregnancy.

To identify genomic regions for short gestation length influenced by different selection events, QP pigs were grouped into three phenotypic gradient groups and compared with 219 other pigs. As the genome regions under selection had decreased genetic diversity, we calculated the genome nucleotide diversity (*π*). There were 2,186 windows with the top 1% absolute diversity ratios in the first population pair (Figure 2A). Among these, 549 windows showed gradient-increased signals in the second and third population pairs. These windows were putative selective regions for short gestation length, and there were 329 candidate genes. In addition, the fixation index (*F*_ST_) was used to measure population differentiation due to the genetic structure. There were 2,190 windows with the top 1% *F*_ST_ values in the first population pair (Figure 2B). Among these, 1,441 windows showed gradient-increased signals in the second and third population pairs. These windows were putative selective regions for short gestation length, and there were 624 candidate genes. Finally, XP-EHH was used to detect selection from the haplotype structure. Two-way top 1% unstandardized XP-EHH value SNPs were chosen in the first population pair (Figure 2C). Furthermore, the gradient-increased signals in the second and third population pairs remained as specific selection signatures for short gestation length. After annotation, we found 1,781 candidate genes. Combining the results of *F*_ST_, *π* ratios, and XP-EHH, 2,512 genes were short gestation length–specific candidate genes (Supplementary Table S5 and Figure 3D). Functional enrichment of these genes showed significant enrichments for GO terms such as the protein tyrosine kinase activity (GO:0004713), cellular response to growth factor stimulus (GO:0071363), and response to growth factor (GO:0070848) and for KEGG pathways such as the MAPK signaling pathway (ssc04010), PI3K-Akt signaling pathway (ssc04151), and ECM-receptor interaction (ssc04512) (Figures 2E,F).

### Differentially Expressed Genes for Short Gestation Length

To identify short gestation length differentially expressed genes, we used LW pigs (a well-known commercial pig breed) with normal gestation length for comparison with QP pigs. Differentially expressed genes were identified between QP pigs and LW pigs in late pregnancy or parturition. A previous study proposed that expression differences between preterm and term birth may be attributable to either preterm pathology or gestational age (Eidem et al., 2016). With the intent of disentangling the effects of gestational age, we excluded differentially expressed genes between late pregnancy and parturition in QP pigs or LW pigs to further distinguish short gestation length genes in late pregnancy or parturition (Figures 3A,D). As a result, 285 annotated genes were differentially expressed in parturition, and 1,190 annotated genes were differentially expressed in late pregnancy for short gestation length. Functional enrichment of the 285 genes showed significant enrichments for GO terms that included leukocyte-mediated immunity (GO:0002443), cAMP-mediated signaling (GO:0019933) and chemokine biosynthetic process (GO:0042033) and for KEGG pathways that included cytokine–cytokine receptor interaction (ssc04060), and cholesterol metabolism (ssc04979) (Figures 3B,C). Functional enrichment of the 1,190 pregnancy genes showed significant enrichments for GO terms that included monocyte extravasation (GO:0035696), cell adhesion (GO:0007155), and regulation of muscle system process (GO:0090257) and for KEGG pathways that included the calcium signaling pathway (ssc04020), MAPK signaling pathway (ssc04010), and PI3K-Akt signaling pathway (ssc04151) (Figures 3E,F). Combining the results from late pregnancy and parturition, 1,185 short gestation length differentially expressed genes were annotated in the ensemble (Supplementary Table S6). Notably, *EGFR* is also a differentially expressed gene in late pregnancy. Interestingly, there were more genes identified in late pregnancy than in parturition. This result indicates that major changes in the expression occurred before the initiation of parturition.

### Candidate Genes for Short Gestation Length

**FIGURE 4:**
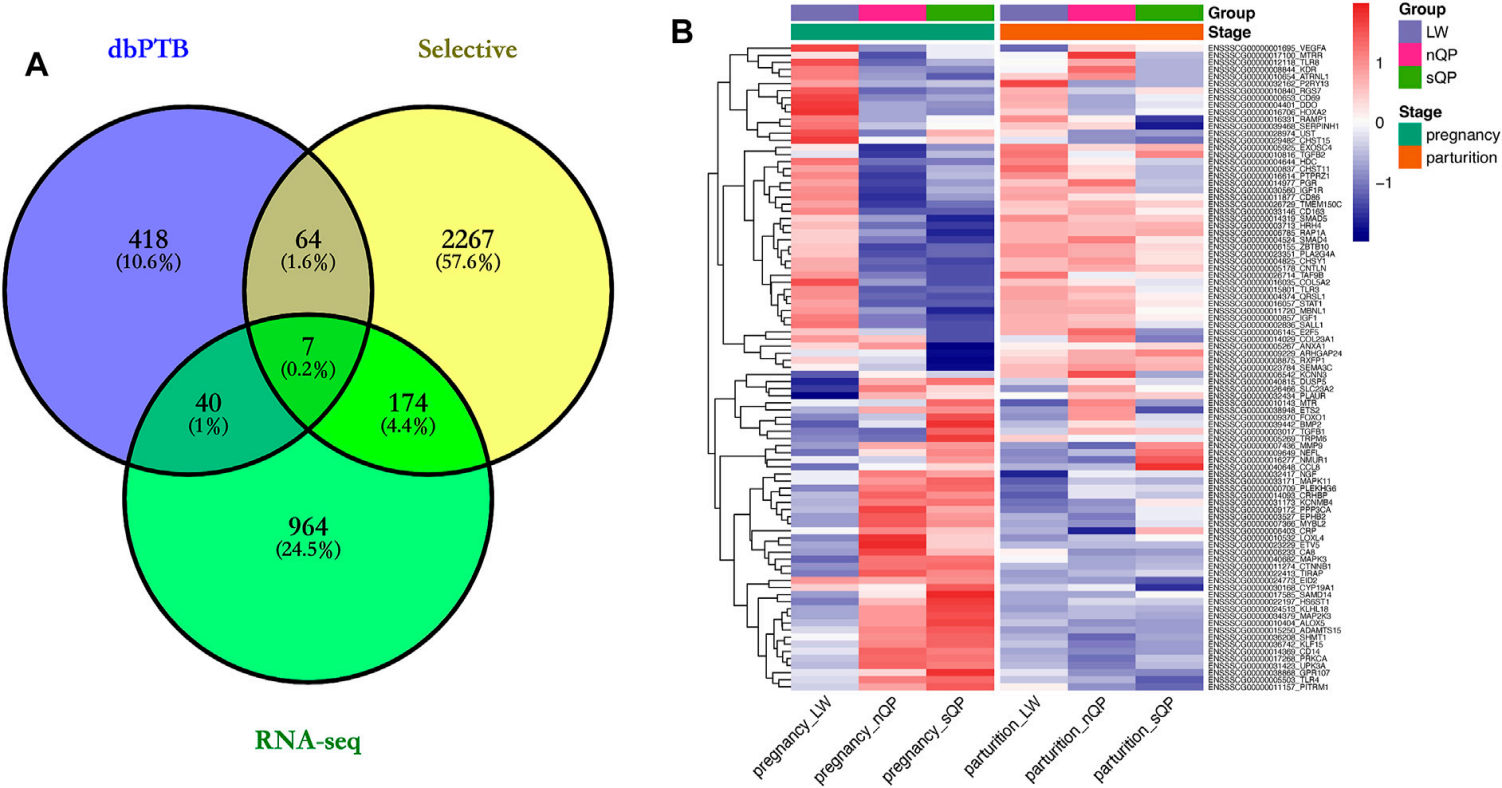
Short gestation length genes. **(A)** VENN plot of selective, RNA-seq candidate genes, and dbPTB genes. **(B)** Expression heatmap of candidate genes in dbPTB.

**FIGURE 5:**
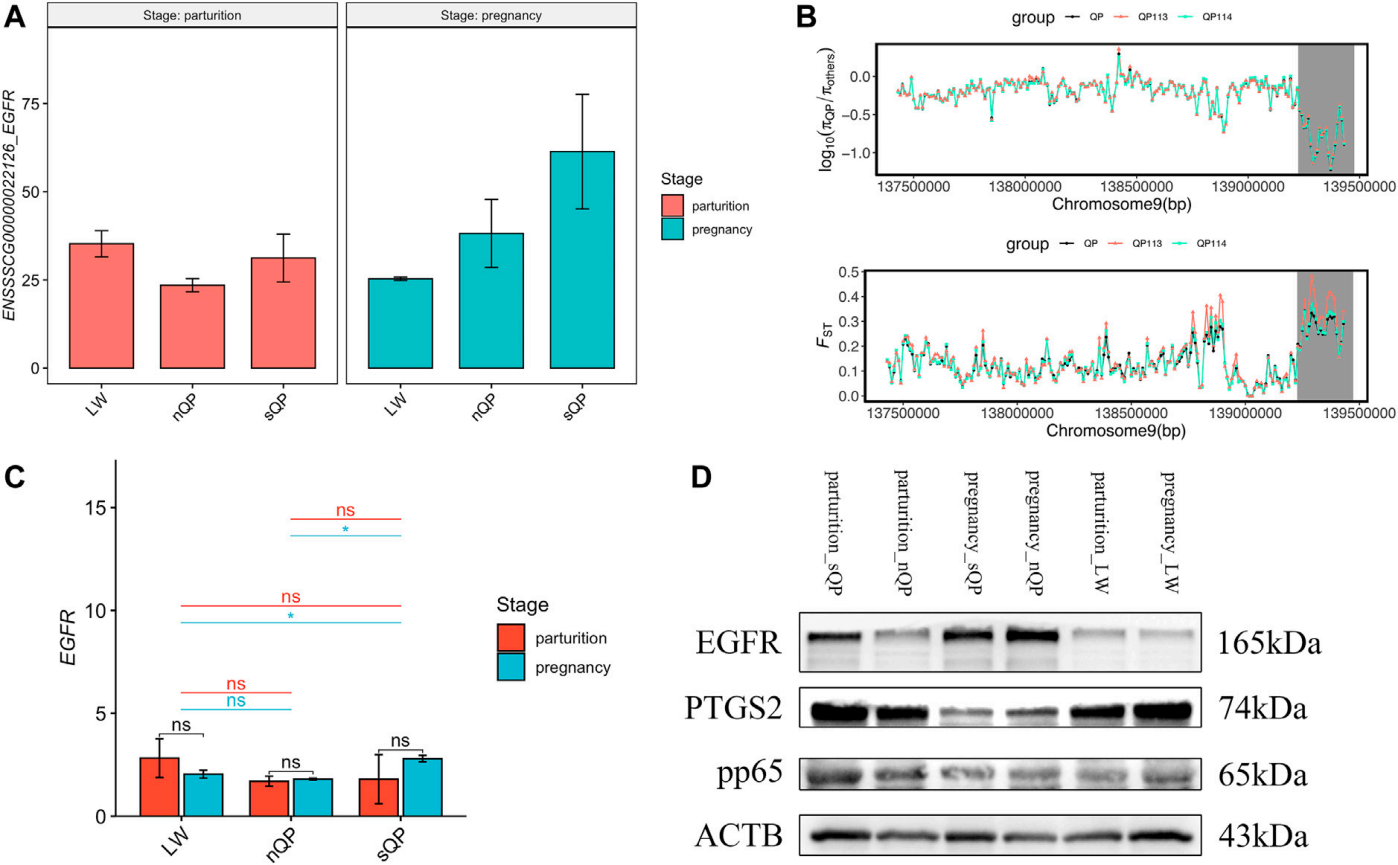
*EGFR* is associated with short gestation length. **(A)** *EGFR* expression in the RNA-seq results. **(B)** Selective sweep analyses of *EGFR*, including *π* and *F*_ST_. **(C)** *EGFR* real-time PCR results of the placenta in QP and LW pigs. **(D)** Western blot results of the placenta in QP and LW pigs.

The list of candidate genes was too large to effectively identify the causal genes. Because short gestation traits are similar to human preterm birth, known preterm birth genes can provide the basis for further identification of short gestation candidate genes. Hence, we compared candidate genes with known preterm birth genes in the preterm birth database to further identify short gestation length genes (Uzun et al., 2012). After this comparison, we identified 111 candidate genes that were reported as preterm birth genes (Supplementary Table S7 and Figure 4A). The heatmap of these genes suggested that there were more differentially expressed genes in late pregnancy (Figure 4B). Additionally, we prioritized the other candidate genes using preterm birth genes as a training gene set. After prioritization, 839 genes were significantly functionally similar to preterm birth genes (Supplementary Table S8). In particular, *EGFR* was the most significant gene (Supplementary Table S8). Interestingly, the RNA-seq results showed a higher expression of *EGFR* in QP pigs with short gestation length in late pregnancy (Figure 5A). Although *EGFR* was not a selective sweep gene for short gestation length, selective sweep analyses still showed higher selective sweeps in the genomic region of *EGFR* than in the adjacent genomic region (Figure 5B). The real-time PCR results of the placenta also exhibited a higher *EGFR* mRNA expression in QP pigs with short gestation length in late pregnancy (Figure 5C). The Western blot results of the placenta also showed increased EGFR protein levels in QP pigs with short gestation length and QP pigs with normal gestation length (Figure 5D). In contrast, Western blot results of the placenta showed that increased *EGFR* expressions in short gestation length QP pigs remained in the parturition stage (Figure 5D). In addition, high levels of the proteins pp65 and PTGS2 also existed in short gestation length QP pigs in parturition (Figure 5D). It is well known that the activation of NF-κΒ and PTGS2 plays key roles in parturition. Therefore, it can be speculated that changes in the *EGFR* expression occurred before parturition and that *EGFR* might play an important role during parturition in QP pigs with short gestation length.

### *EGFR* Regulated NF-κB in pTr Cells

**FIGURE 6:**
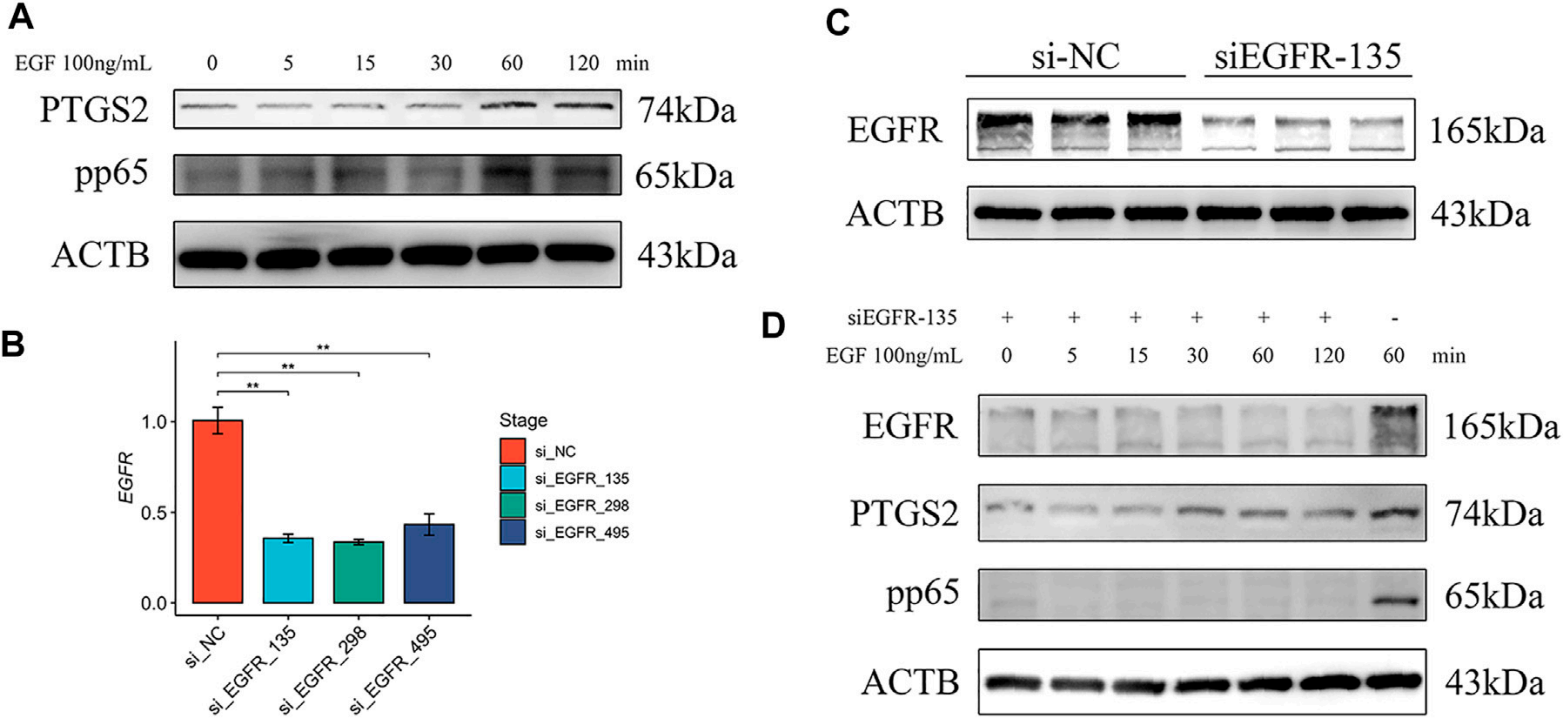
*EGFR* regulated NF-κB and *PTGS2* in pTr cells. **(A)** Western blot results of pTr cells treated with EGF (100 ng/ml) for 0, 5, 15, 30, 60, or 120 min **(B)** *EGFR* real-time PCR results of pTr cells transfected with three siRNAs. **(C)** Western blot results of pTr cells transfected with si_EGFR_135. **(D)** Western blot results of pTr cells treated with EGF (100 ng/ml) for 0, 5, 15, 30, 60, or 120 min after transfection using si_EGFR_135 and treatment with EGF (100 ng/ml) for 60 min.

Functional verification of *EGFR* was subsequently performed in pTr cells. The pTr cells, which participate in placental composition, are essential for pregnancy establishment and maintenance. After treatment with EGF, the Western blot results showed an increased abundance of NF-κB p65 phosphorylation and PTGS2 at 60 min, and this status was maintained until 120 min (Figure 6A). To demonstrate that EGF activated NF-κΒ and *PTGS2* through *EGFR*, we knocked down the expression of *EGFR* using siRNA. *EGFR* mRNA levels in pTr cells were significantly decreased by siRNA (Figure 6B). The Western blot results also showed that EGFR protein levels were reduced by si_EGFR_135 (Figure 6C). Furthermore, EGF was used to treat pTr cells with EGFR knockdown. Notably, the levels of NF-kB p65 phosphorylation and PTGS2 were reduced compared with EGF treatment at 60 min (Figure 6D). These results indicate that the EGF–EGFR system regulated the abundance of NF-kB p65 phosphorylation and PTGS2 in placenta-related cells. NF-κB and *PTGS2* are essential in multiple labor-associated processes (Lappas and Rice, 2007).

## Discussion

In this study, we conducted a study of short gestation length using the whole genome sequencing data of 100 individuals of the QP breed and 219 individuals of other breeds that had been previously published. The PCA and the NJ tree indicated genetic distances between QP and the other breeds. These results might be due to different selective pressures and minimal gene flow. Different selective pressures and minimal gene flow could result in marked morphological and behavioral alterations (Frantz et al., 2015). Selective sweep analyses have been successfully used to detect phenotype-specific selective signals (Ma et al., 2019). Furthermore, RNA-seq is an indispensable tool for transcriptome-wide analysis of differential gene expressions of mRNAs. Therefore, these two approaches can be successfully employed for the identification of candidate genes for short gestation length.

LD decay showed that QP pigs exhibited faster LD decay than the other breeds (Figure 1B). This result suggested that whole genome high-density SNPs were necessary. There was a positive correlation between sequencing depth and the density and quality of called SNPs. Therefore, we used deep sequencing to call SNPs for selective sweep analyses in the present study.

Combining candidate genes based on *F*_ST_, *π*, and XP-EHH, we identified 2,512 candidate genes related to different selective events in QP pigs. Using differentially expressed gene analysis, we identified 1,185 annotated genes in the ensemble for short gestation length. These methods both identified considerable numbers of candidate genes for short gestation length. This result might occur because gestation length is a complex quantitative trait that is usually influenced by multiple genes. Comparing candidate gene sets with known preterm birth genes, we identified 111 candidate genes that were preterm birth genes and 839 new genes that were similar to preterm birth genes. Based on these findings, we speculated that the short gestation length signals might be weak and confusing.

Among the candidate genes, 111 candidate genes were reported as preterm birth genes. Several candidate genes (*FMN1*, *UPK3A*, *KCNMB4*, *UST*, *TMEM150C*, *KLHL18*, *KATNAL2*, *COL23A1*, *TPRG1*, *PITRM1*, *RGS7*, *CA8*, *ZBTB10*, *EXOC4*, *CNTLN*, *HLA-DOB*, *PRL*, *NEGR1*, *HOXA2*, *RAMP1*, *MBNL1*, *NEFL*, *FCER1A*, *HDC*, *DDO*, *QRSL1*, *SALL1*, *TIRAP*, and *CD69*) were differentially expressed in maternal early pregnancy peripheral blood between preterm and term women (Enquobahrie et al., 2009). *PTPRZ1*, *SORCS1*, and *MAPK11* were characterized as myometrial genes initiating preterm labor (Weiner et al., 2010). Variants in *TLR10* were significantly associated with preterm birth in the German population (Heinzmann et al., 2009). In African Americans, mutation in *CD14* was associated with spontaneous preterm birth (Velez et al., 2009). An associated analysis reported that *IGF1R* is a spontaneous preterm birth susceptibility gene (Haataja et al., 2011). *KCNN3* mutation was associated with preterm birth in Argentina (Mann et al., 2012). SNPs in *PRKCA* were associated with preterm birth (Gomez et al., 2010). Preterm premature rupture of membranes is the leading cause of preterm birth. *SERPINH1* encodes heat-shock protein 47 and is essential for collagen synthesis. It has been reported that a functional SNP in *SERPINH1* increases the risk of preterm premature rupture of membranes in African Americans (Wang et al., 2006). Based on these findings, we hold the opinion that short gestation length is similar to preterm birth. Noninfectious and infection-induced models, including in mice, sheep, and monkeys, have been used to investigate the mechanisms that promote preterm birth (Elovitz and Mrinalini, 2004). Mouse models were widely used in investigations of preterm birth (Jaiswal et al., 2015; Huang et al., 2017). However, artificial induction is different from spontaneous preterm birth without induction. Therefore, the QP breed has the potential to be a more fit animal model for investigations of spontaneous preterm birth.

*EGFR* was the most significantly prioritized gene among the novel candidate genes. Verification showed that *EGFR* was highly expressed in the placentas of short gestation length QP pigs and could increase the *PTGS2* expression and activate the canonical NF-κB pathway in pTr cells. A high *EGFR* expression has been suggested to be optimal for epidermal growth factor-induced NF-κB activation (Habib et al., 2001). NF-κB is an important transcription factor family associated with inflammation. Accumulating evidence suggests a role for NF-κB in labor (Lindstrom and Bennett, 2005). IL-1 treatment resulted in the cross-linking of p65 to regions containing NF-κB binding sites on *PTGS2*, and the *PTGS2* expression appears to be regulated by NF-κB (Soloff et al., 2004). *PTGS2* may be more important than *PTGS1* in prostaglandin production and participates in membrane rupture, cervical ripening, inflammation, and myometrial contraction in parturition (Li et al., 2021). Based on these findings, *EGFR* might play an important role in short gestation length through NF-κB and *PTGS2*.

In summary, we determined the genetic architecture of short gestation length *via* multiple strategies using genome and transcriptome sequencing data. These short gestation length candidate genes can be critical resources for understanding short gestation length traits and genetic improvement of gestation length in pig breeding. Experimental verification highlighted that *EGFR* might play an important role in the short gestation length trait due to the induced premature expression of *PTGS2*. These candidate genes still need comprehensive investigation to better understand short gestation length.

## Data Availability

All raw sequences for the 100 QP pigs have been deposited into the NCBI Sequence Read Archive under PRJNA489520. The SNPs have been deposited into the European Variation Archive (https://www.ebi.ac.uk/eva/?Study-Browser) under project PRJEB28579. RNA-seq data in this study have been deposited into the NCBI Sequence Read Archive under PRJNA551700.
